# Total and Plant Protein Consumption: The Role of Inflammation and Risk of Non-Communicable Disease

**DOI:** 10.3390/ijms23148008

**Published:** 2022-07-20

**Authors:** Elena Azzini, Ilaria Peluso, Federica Intorre, Lorenzo Barnaba, Eugenia Venneria, Maria Stella Foddai, Donatella Ciarapica, Francesca Maiani, Anna Raguzzini, Angela Polito

**Affiliations:** CREA-Research Centre for Food and Nutrition, Via Ardeatina 546, 00178 Rome, Italy; ilaria.peluso@crea.gov.it (I.P.); federica.intorre@crea.gov.it (F.I.); lorenzo.barnaba@crea.gov.it (L.B.); eugenia.venneria@crea.gov.it (E.V.); mariastella.foddai@crea.gov.it (M.S.F.); donatella.ciarapica@crea.gov.it (D.C.); francesca.maiani@crea.gov.it (F.M.); anna.raguzzini@crea.gov.it (A.R.); angela.polito@crea.gov.it (A.P.)

**Keywords:** plant protein, NCDs, inflammatory markers, healthy ageing

## Abstract

Background: Inflammatory cytokine levels are associated with Non-Communicable Diseases (NCDs) and can be influenced by a person’s macronutrient profile. This work aims to evaluate the relationship between the compliance with the age-specific recommended protein intake and the levels of inflammatory markers related to the risk of NCDs. Methods: The study participants included 347 participants (119 men and 228 women), ages 18 to 86 years. Cardio-metabolic risk evaluations, including an assessment of the prevalence of Metabolic Syndrome, were performed. Leptin, IL-15, IL-6, and TNF-α levels were measured. Results: The adequacy of the total protein (TP) intake was lower in old people compared to individuals aged <60 years, and only few volunteers consumed the suggested 50% plant protein (PP) for a healthy and sustainable diet. A lower risk of NCDs with a PP consumption above at least 40% was observed only in old individuals. A differential effect on TNF-α and IL-6 was observed for both TP and PP intake by gender and age class, whereas for leptin and IL-15 only significant interactions among sex and the class of age were found. Conclusion: Although our data suggest that consuming more than 40% of PP can reduce the risk of NCDs, the effect of gender differences on cytokine levels should be considered in larger studies.

## 1. Introduction

The latest findings from the Global Burden of Disease Study provide insights on the increased risk of serious illness and death from COVID-19 associated with several risk factors and non-communicable diseases (NCDs), including obesity, diabetes, and cardiovascular disease (CVD) [1]. Over the past 30 years, the largest absolute increases in health loss have occurred worldwide, affecting particularly older adults including ischemic heart disease (increasing by 50% between 1990 and 2019), diabetes (up 148%), and stroke (32%). Collectively, in 2019, metabolic risks (including obesity, high blood sugar, high blood pressure, and high cholesterol) accounted for nearly 20% of total health loss (50% higher than in 1990: 10.4%). As widely reported in the scientific literature, low-grade inflammation is often associated with the appearance of NCDs, and the expression and secretion of inflammatory biomarkers can be influenced by the macronutrient profile. Immune-senescence and inflammaging are considered key features of the frail elderly, influencing their susceptibility to infections and worsening disease progression [2,3]. It has been suggested that a supplementation with amino acids could enhance the immune status, thereby reducing the morbidity and mortality of infectious diseases for individuals with malnutrition [4]. The quantity and quality of protein are different in plant- and animal-based foods. Animal protein (AP) provides all the essential amino acids and is more bioavailable compared with their plant counterpart [5]. However, several epidemiological studies have shown an inverse association between high intakes of plant protein (PP) and cardiometabolic risk and mortality [6]. Accordingly, unhealthy and unsustainable diets have been recognized as strongly contributing to the escalating burden of diet-related NCDs, aside from adversely impacting the local food systems and natural environment [7,8]. Population reference intakes for total protein (TP) are age specific and apply to mixed dietary protein from both animal and plant sources [9,10]. Moreover, it has been pointed out that a high intake of proteins is necessary for elderly adults with sarcopenia or for patients with severe illness [11]. Aside from the role of genetics and environmental exposure, the dominant risk factors for non-communicable diseases are behavioral, and by and large, they could be avoided and prevented (or at least attenuated) over the course of one’s life via lifestyle modifications and can thus reduce their great economic burden. 

This work aimed to evaluate the linkage between compliance with protein intakes and the levels of inflammatory markers related to the risk of NCDs within an Italian population group. 

## 2. Results

The total sample included 119 males and 228 females aged 57.4 ± 15.8 and 57.7 ± 15.2 years, respectively. According to the study aim, the volunteers were grouped by sex, age class, and the quintile (Q) category of TP consumption (g/kg/d). The mean Body Mass Index (BMI) of the participants <60 years old (26.8 ± 5.0 kg/m^2^) evidenced a statistically significant lower BMI with respect to those ≥60 years old (28.5 ± 4.2 kg/m^2^) (*p* = 0.001) (Supplementary Table S1). 

Table 1 shows the intake of animal (AP) and plant protein (PP) as g/kg/d, fat (FAT), and carbohydrates (CHO) expressed as a percentage of energy (%En) and the daily consumption of fiber (g/d). A statistically significant difference (*p* = 0.035) was found in PP intake (g/kg/d) among the different levels of age class (adult vs older). No differences by age class were observed, while multiple comparison procedures (Bonferroni t-test) evidenced a statistical difference (*p* < 0.05) in the PP mean values among the different quintile categories of average protein intake (g/kg/d) (Table 1). AP consumption (g/kg/d) presented a statistically significant (*p* < 0.05) interaction between sex and age class upon the quintiles of average protein intake (g/kg/d). All pairwise multiple comparison procedures (Bonferroni t-test) found a significant interaction (*p* = 0.002) by sex (men vs women) within the Q2–Q5 categories of average protein intake as well as by age class (adults vs. older) within the Q1–Q5 categories of average protein intake (*p* < 0.001). On average, intakes of CHO were within the dietary guidelines (ranged from 45% to 60%), whereas both younger and older individuals showed a %En from fat above the dietary recommendations (20–35%). On the contrary, the fiber intake was significantly different among the quintiles of average TP intake (Table 1), and the mean values were all under the dietary guidelines (at least 25 g/d).

**Figure 1:**
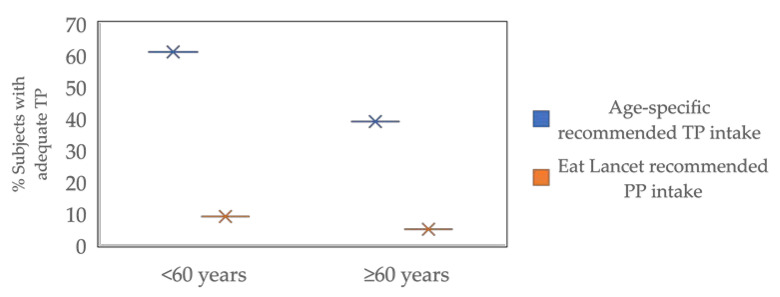
Percentage of individuals with adequate protein intake. TP: total protein (LARN age-specific recommendations), PP: plant protein.

The percentage of individuals with TP consumption in line with the age-specific reference values of protein intake for an Italian population (0.9 mg/kg and 1.1 mg/kg for young and old people, respectively) presented age differences (Figure 1).

For 221 adults (68 M and 153 F), 61% consumed a total daily protein amount in line with the recommendation (≥0.90 g/kg/d), while only 39% of the 126 older adults (51 M and 75 F) had a daily TP intake in line with the suggested dietary target recommendation (≥1.1 g/kg/d). Moreover, among the subjects consuming the recommended daily protein intake, only 9% of adults consumed at least 50% from PP, as suggested by the sustainable eating model recommended by EAT Lancet [10], and only 5% of older adults reached this goal (Figure 1). 

**Figure 2:**
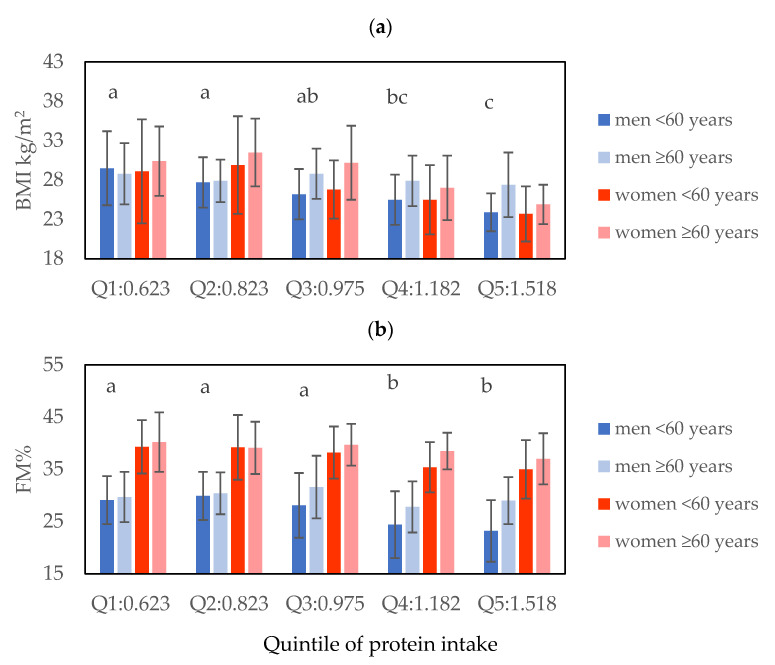
Body mass index (kg/m^2^) (**a**) and fat mass percentage (FM%) (**b**) by quintiles of protein intake. Three-way ANOVA significant differences (*p* < 0.05) between different letters (**a**) BMI kg/m^2^ = body mass index; Q1 vs. Q5 (*p* < 0.001); Q1 vs. Q4 (*p* = 0.004); Q2 vs. Q5 (*p* < 0.001); Q2 vs. Q4 (*p* = 0.009); Q3 vs. Q5 (*p* = 0.003). (**b**) FM% Q2 vs. Q5 (*p* = 0.004), Q2 vs. Q4 (*p* = 0.021), Q1 vs. Q5 (*p* = 0.004), Q1 vs. Q4 (*p* = 0.025), Q3 vs. Q5 (*p* = 0.010), and Q3 vs. Q4 (*p* = 0.050).

The mean BMI values among the different protein intake quintiles were statistically higher at lower quintiles (Figure 2a) and the BMI of the participants <60 years old (26.8 ± 5.0 kg/m^2^) was statistically significantly lower compared to those ≥60 years old (28.5 ± 4.2 kg/m^2^) (*p* = 0.001) (Supplementary Table S1). As expected, the mean estimates of Fat Mass (%) were statistically different (*p* ≤ 0.001) among sex and age class (Supplementary Table S1). Moreover, FM% was higher in lower quintiles of average protein intake (Figure 2b). The same trend was observed in the mean fat-free mass (FFM) among the different levels of sex, age class, and quintiles of protein (Supplementary Table S1). 

Insulin resistance, assessed as the homeostasis model assessment-estimated insulin resistance (HOMA-IR), highlighted a statistically significant interaction between sex and age class upon different TP quintiles (*p* = 0.001) (Supplementary Table S1). We found a significant sex and age class interaction at the level Q1 category of average protein intake (g/kg/d) (*p* = 0.003). A significant difference in the HOMA-IR mean values (*p* = 0.025) among the different levels of age class evaluated within the level Q3 category of average protein intake (averaging over levels of sex) was observed.

**Figure 3:**
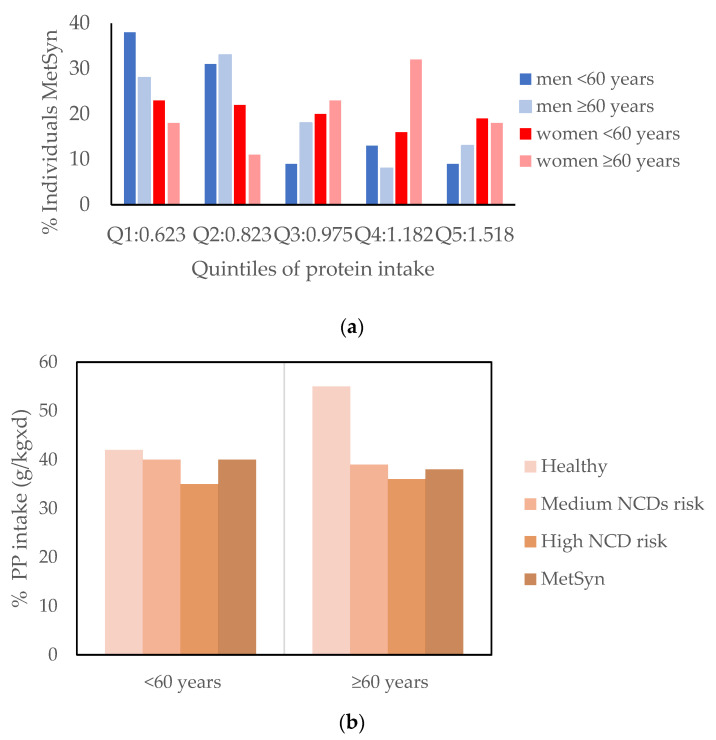
(**a**) Prevalence of MetSyn by quintile of protein intake. Difference of prevalence of MetSyn by quintiles of protein intake explore by Chi-square = 29.574 (*p* ≤ 0.001). (**b**) % of PP intake on total protein (g/kg/d) by age class and disease risk factors by health outcomes and age class.

Of the total sample, only 51 subjects (13 M and 38 F) did not present any disease risk factor (Supplementary Table S2). The prevalence of hypertension was significantly higher in men than women (*p* < 0.05), whereas in older subjects the prevalence of the risk of NCDs (including a disease risk for type 2 diabetes, hypertension, and CVD), hypertension, and dyslipidemia were significantly higher (*p* < 0.05, Supplementary Table S2). These outcomes were significantly different among the quintiles of protein intake (Supplementary Table S2). From that, differences in the metabolic syndrome (MetSyn) (a cluster of disease risk factors for CVD) prevalence among the quintiles of protein intake were found (Figure 3): as the consumption of TP increased, the prevalence of MetSyn tended to decrease (*p* < 0.05).

When observing the distribution of the PP intake (g/kg/d) expressed as % TP consumption (g/kg/d) by age class and the presence of MetSyn and the risk of NCDs, the consumption of PP above 40% of TP showed a protective effect only in old individuals (Figure 3b). 

Table 2 and Table 3 show the distribution of adipokine leptin and cytokines by sex, age class, and the quintile category of average protein intake (g/kg/d) and the correlations between the parameters of interest. Obviously, being produced by FM, a statistically significant difference (*p* = 0.001) was revealed by sex in the leptin circulating levels. Despite this, in the overall sample, leptin resulted in being inversely correlated with PP and fiber intakes, probably due to the high inverse correlation observed between BMI and these plant foods components (Table 3). Moreover, leptin was correlated with the adipo-myokines TNF-α and IL-6, but not with IL-15 (Table 3), probably produced from muscle mass rather than from fat mass.

On the other hand, TNF-α and IL-6 were also markers of low-grade systemic inflammation and were correlated in our population (Table 3). Concerning these inflammatory cytokines, a statistically significant interaction among sex, age class, and the quintiles of TP was found (Table 2). The lower and higher TNF-α mean concentrations were found in women < 60 years—Q4 TP intake (1.05 ± 0.16 pg/mL) and men ≥ 60 years—Q1 TP intake (2.53 ± 0.25 pg/mL), respectively (Table 2). On the contrary, the lower mean level of IL-6 (1.03 ± 0.61 pg/mL) was found in men < 60 years—Q2 TP intake and the higher mean level (7.35 ± 0.65 pg/mL) in women ≥ 60 years—Q5 TP intake (Table 2). Moreover, TNF-α was inversely correlated with PP intake (Table 3). 

Regarding the cytokine IL-15, no gender and age differences were found among the quintiles, but we found a statistically significant interaction between sex and age class (*p* = 0.018), with major differences between men (5.35 ± 0.66 pg/mL) and women (3.58 ± 0.21 pg/mL) within the age class ≥60 years and the Q4 TP (Table 2), whereas no relationship was found with PP and fiber intakes, as well as with other cytokines, leptin, and BMI (Table 3). As reported in Table 3, IL-15 was only related to age, but the correlation was greater for TNF- α and IL-6. 

**Figure 4:**
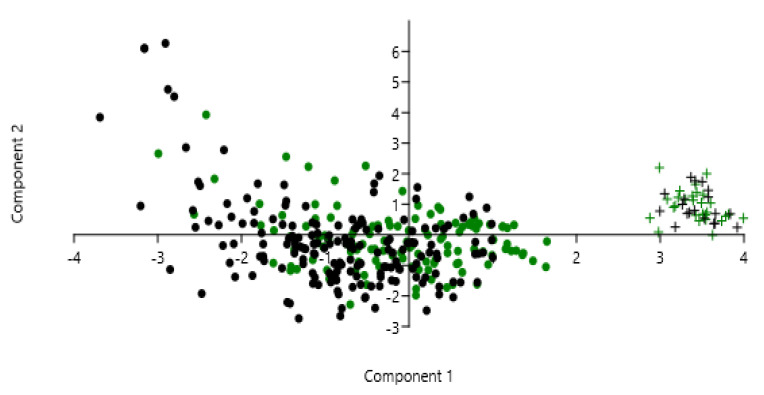
Principal component analysis of macronutrient dietary pattern relating to consumption, biochemical markers, and healthy outcomes: • = unhealthy ≥ 60 years; **+** = healthy ≥ 60 years; • = unhealthy <60 years; **+** = healthy <60 years.

The relationship between dietary intake and health is very complex to study because the dietary exposure comprises a lot of different nutrients and other bioactive constituents acting synergistically as well as in opposition with each other and their interactions cannot be understood by studying the effects of single dietary components. Therefore, to identify associations among the studied biochemical variables, the macronutrient dietary pattern relating to consumption and healthy outcomes was carried out using a multivariate analysis by principal component analysis. In our study, the PC1, PC2, and PC3 represent 21.45%, 14.53%, and 12.87% of the system variance, and based on eigenvalues >1.5 the 76.74% of variance was reached and enables us to discriminate the health of younger and older subjects (Figure 4) based on dietary pattern, biochemical markers of inflammation, and the presence of unhealthy conditions.

## 3. Discussion

Our work examined the dietary intake and biological markers of inflammation related to the risk of NCDs in an Italian population group. Although our results concerned an observational study conducted on a population group living in the center of Italy and thus could not be generalized to all, we were able to identify some linkages among different health outcomes and prevailing eating habits. The first and most important consideration is about the studied population: we found only 15% healthy subjects. Significant differences in the consumption of AP and PP such as fiber intake by sex, age class, and different quintiles of average protein intake (g/kg/d) were found. In healthy subjects, the preference towards plant protein was prevalent and this diversity of the protein fraction could make the difference concerning the different compositions of amino acids, in addition to suggesting that the consumption of vegetable proteins provides other specific non-protein compounds of nutritional interest and potential beneficial effects [12]. The human body is made up of thousands of different proteins, each with a specific function. Proteins set up the structural components of our cells and tissues as well as many enzymes, hormones, and the active proteins secreted from immune cells. Proteins are essential for life because they supply the essential amino acids needed for the growth and maintenance of our cells and tissues, and their requirements in turn depend on our stage of life. If people consume a varied diet with respect to the total amount of protein that meets their daily needs, the quality and digestibility of the eaten proteins should not be a concern [13]. Our data indicate that the PP consumption above 40% of one’s total daily amount is protective against metabolic risks, despite Li and Wu’s research [14], which indicated the pivotal role of functional AA (e.g., arginine, cysteine, glutamate, glutamine, glycine, taurine, and tryptophan) and glutathione, abundant in animal-sourced foodstuffs, for optimizing immunity and health in humans. As people eat foods and not nutrients, we should choose protein-rich foods that not only provide essential amino acids but also support a healthy and sustainable diet. In fact, consumption patterns depend on income, prices, individual preferences and beliefs, cultural traditions, as well as geographical, environmental, social, and economic factors. The COVID-19 health emergencies and the failure to avoid the increase of chronic diseases by preventing risk factors have a devastating result on vulnerable populations, and as is well-known, protein-related nutritional deficiencies cause fragility and make individuals more susceptible to certain diseases, particularly infectious diseases, such as pneumonia in the cold seasons. Even though some diet–disease relationships are difficult to identify within a single population, diet may strongly influence the average risk of disease occurrence within that population. Moreover, studying global diet markers instead of investigating isolated nutrients helps to understand the factors that contribute to NCDs, thus enabling an investigation of the association between dietary patterns and the indices that evaluate the quality of the diet with inflammatory markers. There is a large consensus that healthy dietary patterns exhibit an inverse relationship with the circulating levels of inflammatory biomarkers [15]. A high intake of vegetables, whole grain cereals, fruits, chestnuts, fish, and olive oil together with a low consumption of meat, sugary drinks, processed foods, and saturated fat represent a dietary pattern capable of counteracting the inflammatory state and reducing the risk of NCDs [16]. Additionally, the daily consumption of fruits and vegetables in an adequate amount can reduce oxidative stress and low-grade inflammation markers [17]. We found a significant positive correlation between PP and fiber consumption, both negatively correlated with circulating leptin levels (*p* < 0.05). Furthermore, the same trend, not statistically significant, was observed in the average circulating leptin levels in the adult groups and older women according to protein quintiles, with leptin tending to decrease as the quintile of protein consumption increases. A systematic review and meta-analysis of randomized controlled trials studied the effect of dietary fiber on serum leptin level and found that dietary fiber could lower serum leptin levels in an obese sample [18]. Circulating levels of leptin were significantly higher in subjects with a high risk of CV and who were affected by MetSyn and hypertension. An expert opinion elucidating the different impacts of animal vs. plant protein towards modifying the cardiometabolic risk factors by observational and interventional studies confirmed that increasing protein intake, especially plant-based proteins, and certain animal-based proteins (poultry, fish, unprocessed red meat low in saturated fats, and low-fat dairy products) have a positive effect [19]. Our findings indicated a positive significant correlation among circulating levels of IL-6 with TNF-α (*p* < 0.05) as well as a tendency towards higher values for IL-6 and TNF-α in older adults. Several studies reported that the age-related NF-κB-signaling upregulation of proinflammatory gene expression could lead to a release of proinflammatory mediators called the senescence-associated secretome, particularly IL-6, which we also found to be remarkably higher, and in TNF-α to a lesser extent [20]. In addition, Franceschi et al. reported that unpredictable, persistent, and increasing evolutionary exposure to a variety of external and internal stressors that occur with age activates innate and adaptive immune pathways involved in the inflammatory response, which also involves muscle and adipose tissue [21]. Older people often have multimorbidity and frailty associated with inflammation. Recent data suggest that inflammation is fueled by a variety of gut microbiota-derived stimuli and by the continued production of potentially inflammatory molecules released/secreted as a consequence of cell death and organelle dysfunction [22]. These physiological and vital phenomena for survival undergo a progressive increase with age until exerting age-related pathologies. In a systematic review on the effect of nutrition on aging, Leitão et al. demonstrated the necessity of improving dietary patterns to reducing the emergence and development of several comorbidities, to promoting healthy aging, and increasing life expectancy as well as dietary patterns’ importance to the development of nutrition interventions that target aging-related biomarkers [23]. By principal component analysis, we were able to assess and demonstrate the interconnection between nutrients, biochemical markers, and health. Our data emphasize the strict relationship between consuming at least 40% of plant protein intake and health outcomes in both adults and older adults than adequate intake. Recently, Simonson et al. underlined the potential therapeutic effect of tuning the quality and quantity of proteins or even specific amino-acids on the body composition and metabolic syndrome parameters of obese patients [24]. Our data indicated the same trend in both age classes, where an increasing quintile of protein intake corresponds to a decrease in HOMA-IR value. In line with the suggestion of Day et al., there is a need for further research and innovation to enhance and explore the nutritional quality and healing potential of plant proteins and to focus on the emerging technologies for improving their bioavailability, digestibility, and organoleptic properties [25]. As recently reported by Branca and colleagues, the United Nations Decade of Action on Nutrition, along with the 2030 Sustainable Development Agenda and Goals, represent a great opportunity to cost-effectively improve diets, eliminate malnutrition, reduce death and disability from NCDs, and promote sustainable development in addition to mitigating the threats posed by future pandemics to vulnerable populations [26].

## 4. Materials and Methods

### 4.1. Study Population 

The sample was recruited from March 2015 to October 2016 in a central geographic area of Italy in the areas of Lazio and Abruzzo. The data were collected from July 2015 to December 2016. Study participants included 383 free-living volunteers (136 males and 247 females)**,** aged 18 to 86 years. A complete clinical check-up including a full medical assessment was performed on all subjects, as well as an assessment of drug use and blood pressure. The volunteers were selected based on the absence of pathologies potentially interfering with the parameters studied, and the absence of ascertained viral infections, allergies, and food intolerances. Subjects who did not meet these criteria were excluded. Among the recruited subjects, a high percentage of male volunteers chose not to join the study. 

After screening and the acceptance of blood withdrawal, 347 participants (119 men and 228 women) were considered eligible. The study was conducted in accordance with the Declaration of Helsinki on the performance of human trials, and participants provided informed consent. The Ethics Committee of Lazio 2 approved the procedure as well as the performance of this study. 

### 4.2. Data Collection

All the enrolled volunteers’ food consumption, anthropometric measurements (weight, height, and circumferences), and body compositions were evaluated. In addition, blood sampling was carried out. 

### 4.3. Food Consumption Assessment

The food consumption was determined by a validated food diary on four consecutive days including the weekend. All consumed foods and beverages were recorded by participants and the day after a dietician verified and checked the registration’s propriety. Furthermore, to improve the accuracy on the estimation of the portions, a photo album was used [27]. To calculate energy, macro and micronutrients from daily consumption Italian Food Composition Tables were used [28].

According to the protein consumption pattern of our sample and The Dietary Reference Values of Nutrients and Energy for the Italian population (LARN) of 0.90 and 1.1 (g/kg/d) for adults and elderly, respectively, quintile categories of averaged protein intake (g/kg/d) were calculated and resulted as follows: Q1:0.623, Q2:0.823, Q3:0.975, Q4:1.182, and Q5:1.518 [29]. 

### 4.4. Anthropometric Measurements

Anthropometric measurements, including body weight, stature, circumferences, and skinfold thicknesses, were assessed in accordance with the techniques described by Lohman et al. [30]. Body weight was recorded to the nearest 0.01 kg using a calibrated digital balance (SECA 821); height was measured to the nearest 0.1 cm with a wall stadiometer (SECA 225). The BMI was calculated by dividing the subject’s weight in kilograms by the square of their height in meters (kg/m^2^) and subjects were classified into three groups according to WHO criteria [31]. Waist and hip were measured using a flexible inelastic tape to the nearest 0.1 cm. Waist circumference alone or in relation to hip circumference was used as an index of the distribution of adipose tissue and visceral obesity to obtain indicators of NCD risk factors.

FM and FFM were derived by four skinfold thickness (biceps, triceps, subscapular, and suprailiac) using Durnin and Womersley’s equations [32]. Body fat was calculated using Siri’s formula [33]. Skinfold thicknesses were measured on the nondominant side in triplicate to the nearest 0.2 mm with a calibrated Harpenden caliper according to the standard procedure [30]. All measurements were made in each center by the same skilled observer. Standard error of measurements ranged from 0.27–0.56 mm for skinfold thickness

### 4.5. Systolic and Diastolic Blood Pressure

Systolic and diastolic blood pressure was measured in mm Hg using Omron M6 (HEM-7001-E)**,** after subjects had been resting for approximately 8–10 min.

### 4.6. Blood Collection and Analysis

Blood samples were drawn from the right antecubital vein after an overnight fast and collected in EDTA- or heparin- containing tubes. The plasma was stored at −80 °C until analysis. The assessments included a serum lipid profile (total cholesterol, high-density lipoprotein cholesterol (HDL-C), low-density lipoprotein cholesterol (LDL-C), and triacylglycerol concentrations), glucose, insulin, leptin, IL-15, IL-6, and TNF-α levels. Clinical chemistry determinations (ClinChem Control 1; Sentinel Diagnostics, Milan, Italy) were performed using enzymatic tests (Sentinel Diagnostics, Milano, Italy). Insulin levels were measured by enzyme-linked immunosorbent assay commercial kits (DRG International, Inc., Springfield, NJ, USA; BD Biosciences ELISA reagent, San Jose, CA, USA; and Boster Biological Technology, Ltd., Pleasanton, CA, USA). Plasma cytokine levels were assessed using multiplex bead assay (Bio-Rad Laboratories, Hercules, CA, USA and EMD, Bioscience Research Reagents, Temecula, CA, USA).

### 4.7. Cardio-Metabolic Risk Evaluations

The HOMA-IR was used to assess insulin resistance. HOMA-IR was calculated by multiplying fasting plasma insulin (mU/L) by fasting plasma glucose (mg/dL) and then dividing by the constant 405, and a HOMA-IR value > 2.5 was considered to be indicative of insulin resistance [34,35]. 

Risk of NCDs (Disease risk for type 2 diabetes, hypertension, and CVD); medium risk presence of BMI 25.0–29.9 kg/m^2^ and waist circumference (≥88 cm women. ≥102 cm men); high risk presence of BMI ≥ 30 kg/m^2^ and waist circumference (≥88 cm women. ≥102 cm men) [36].

MetSyn was defined as having at least three of the following five components: elevated waist circumference (≥88 cm women. ≥102 cm men), elevated triglycerides (≥150 mg/dL) or drug treatment for hypertriglyceridemia, low HDL cholesterol (<40 mg/dL male; <50 mg/dL female), high blood pressure (systolic ≥ 130 mm Hg or diastolic ≥ 85 mm Hg) or hypertensive drug treatment, and high fasting glucose (>100 mg/dL) or drug treatment for hyperglycemia [37]. 

Dyslipidemia was defined as total cholesterol (≥200 mg/d), hypertriglyceridemia (≥150 mg/dL, Low HDL cholesterol (<40 mg/dL male; <50 mg/dL female), or antilipidemic treatment [38]. 

Hypertension was defined as systolic pressure ≥ 130 mm Hg or diastolic pressure ≥ 85 mm Hg) or hypertensive drug treatment [39]

### 4.8. Statistical Analysis 

Data are presented as mean ± standard deviation and mean ± sem. Normality tests were performed using the Kolmogorov–Smirnov test. Pearson’s correlation was used to determine the association between continuous variables and multiple comparison was performed by ANOVA with post hoc correction by Bonferroni test. χ^2^ test was also applied (StatSoft^®^ STATISTICA 8 for Windows (StatSoft. Italia Srl, Vigonza, Italy). Values of *p* < 0.05 were considered statistically significant. Principal component analysis (PCA) was performed to evaluate the variables’ relationships among the study subjects. PCA was performed based on the individual dietary patterns, biochemical parameters, and health outcomes, and based on eigenvalues >1.0 the extracted factors explained 76.74% of variance using PAST (PAleontological STatis-tics).

## Figures and Tables

**Table 1:** Macronutrients and fiber intake by sex, age class, and quintile category of average protein consumption.

Quintiles of Protein Intake by Sex and Age	AP g/kg/d	PP g/kg/d	%En FAT	%En CHO	FIBER g/d
Men < 60 years (overall)	0.62 ± 0.26	0.39 ± 0.15 *	36.80 ± 6.45	48.65 ± 7.97	16.67 ± 8.39
Q1:0.623 (g/kg/d)	0.29 ± 0.15 ^a^	0.32 ± 0.16 ^a^	36.67 ± 8.31	50.30 ± 11.30	13.57 ± 6.06 ^a^
Q2:0.823	0.5 ± 0.1 ^b^	0.33 ± 0.10 ^a^	35.81 ± 7.44	48.94 ± 8.39	13.58 ± 4.79 ^a^
Q3:0.975	0.63 ± 0.12 ^c^	0.34 ± 0.10 ^ab^	39.35 ± 6.14	46.52 ± 7.01	13.02 ± 5.37 ^a^
Q4:1.182	0.74 ± 0.11 ^d^	0.47 ± 0.07 ^b^	36.87 ± 4.99	48.30 ± 5.48	22.73 ± 12.06 ^bc^
Q5:1.518	0.94 ± 0.26 ^e^	0.55 ± 0.19 ^c^	35.30 ± 3.97	49.17 ± 5.13	20.78 ± 5.94 ^bc^
Men ≥ 60 years (overall)	0.71 ± 0.31	0.33 ± 0.10 **	36.70 ± 6.75	45.37 ± 7.21	15.26 ± 5.94
Q1:0.623 (g/kg/d)	0.33 ± 0.11 ^a^	0.27 ± 0.06 ^a^	38.05 ± 7.34	47.78 ± 7.93	13.62 ± 6.65 ^a^
Q2:0.823	0.49 ± 0.08 ^b^	0.34 ± 0.09 ^a^	36.70 ± 6.97	47.61 ± 8.04	13.82 ± 5.96 ^a^
Q3:0.975	0.64 ± 0.10 ^c^	0.32 ± 0.09 ^ab^	38.98 ± 7.23	42.66 ± 7.04	15.52 ± 5.28 ^ab^
Q4:1.182	0.83 ± 0.09 ^d^	0.38 ± 0.12 ^b^	34.28 ± 6.31	43.91 ± 4.28	16.59 ± 3.98 ^ab^
Q5:1.518	1.24 ± 0.21 ^e^	0.43 ± 0.12 ^c^	35.51 ± 5.09	44.89 ± 4.86	20.08 ± 4.91 ^bc^
Women < 60 years (overall)	0.63 ± 0.29	0.40 ± 0.16 *	38.70 ± 6.44	47.53 ± 7.43	14.43 ± 5.99
Q1:0.623 (g/kg/d)	0.32 ± 0.14 ^a^	0.31 ± 0.13 ^a^	39.28 ± 5.76	49.93 ± 6.95	12.45 ± 6.96 ^a^
Q2:0.823	0.53 ± 0.09 ^b^	0.28 ± 0.10 ^a^	41.74 ± 7.69	43.76 ± 9.00	13.74 ± 6.24 ^a^
Q3:0.975	0.57 ± 0.12 ^bc^	0.41 ± 0.11 ^a^	37.32 ± 6.62	49.59 ± 7.94	13.88 ± 6.10 ^a^
Q4:1.182	0.73 ± 0.18 ^cd^	0.44 ± 0.17 ^ab^	36.27 ± 5.82	48.73 ± 6.60	15.03 ± 5.12 ^ab^
Q5:1.518	1.03 ± 0.20 ^de^	0.52 ± 0.15 ^c^	38.89 ± 5.30	46.35 ± 5.27	16.24 ± 5.38 ^ab^
Women ≥ 60 years (overall)	0.64 ± 0.28	0.39 ± 0.14 **	38.01 ± 6.99	46.65 ± 7.12	16.34 ± 6.97
Q1:0.623 (g/kg/d)	0.29 ± 0.12 ^a^	0.32 ± 0.12 ^a^	36.77 ± 7.81	50.72 ± 8.59	15.09 ± 8.97 ^ab^
Q2:0.823	0.54 ± 0.06 ^b^	0.27 ± 0.05 ^a^	37.11 ± 7.08	46.42 ± 6.21	12.89 ± 4.33 ^a^
Q3:0.975	0.59 ± 0.11 ^bc^	0.39 ± 0.11 ^ab^	38.50 ± 7.15	46.32 ± 7.33	16.56 ±5.33 ^ab^
Q4:1.182	0.75 ± 0.13 ^cd^	0.41 ± 0.13 ^b^	39.49 ± 5.99	45.49 ± 6.99	15.98 ± 7.15 ^ab^
Q5:1.518	1.02 ± 0.28 ^de^	0.52 ± 0.14 ^c^	38.19 ± 7.80	44.28 ± 5.23	20.19 ± 7.26 ^bc^

AP = Animal protein; PP = Plant protein; En= energy; CHO = Carbohydrate; FAT = Lipid. Three-way ANOVA Factor by quintiles ^abcde^: significant differences (*p* < 0.05) between different letters; by age class (* vs. **).

**Table 2:** Concentration of leptin and cytokines by sex, age class, and quintile category of average protein consumption.

Quintiles of Protein Intake by Sex and Age	Leptin pg/mL	TNF-α pg/mL	IL-6 pg/mL	IL-15 pg/mL
Men < 60 years (overall)	6414 ± 2755	1.31 ± 0.11 *	1.30 ± 0.30 *	4.05 ± 0.18
Q1:0.623 (g/kg/d)	8093 ± 5719	1.12 ± 0.23 ^a^	1.48 ± 0.63 ^ab^	4.59 ± 0.38 ^bc^
Q2:0.823	9899 ± 5537	1.28 ± 0.21 ^ab^	1.03 ± 0.61 ^a^	4.33 ± 0.37 ^ab^
Q3:0.975	6831 ± 7004	1.55 ± 0.28 ^b^	1.73 ± 0.77 ^bc^	3.79 ± 0.46 ^a^
Q4:1.182	5967 ± 5119	1.37 ± 0.23 ^ab^	1.04 ± 0.63 ^a^	3.94 ± 0.38 ^ab^
Q5:1.518	3280 ± 6678	1.23 ± 0.27 ^ab^	1.22 ± 0.74 ^ab^	3.59 ± 0.44 ^a^
Men ≥ 60 years (overall)	10,124 ± 3380	2.05 ± 0.14 **	2.70 ± 0.37 **	4.65 ± 0.22
Q1:0.623 (g/kg/d)	11,159 ± 6143	2.53 ± 0.25 ^c^	2.27 ± 0.68 ^bc^	4.47 ± 0.40 ^bc^
Q2:0.823	8453 ± 5919	1.82 ± 0.24 ^bc^	2.73 ± 0.65 ^bc^	4.25 ± 0.39 ^bc^
Q3:0.975	11,424 ± 6678	2.14 ± 0.27 ^bc^	3.24 ± 0.73 ^c^	5.19 ± 0.44 ^c^
Q4:1.182	7700 ± 9905	2.00 ± 0.40 ^bc^	1.75 ± 1.09 ^bc^	5.35 ± 0.66 ^c^
Q5:1.518	11,889 ± 8371	1.76 ± 0.34 ^bc^	3.48 ± 0.92 ^c^	3.97 ± 0.56 ^ab^
Women < 60 years (overall)	26,698 ± 1812	1.25 ± 0.07 *	1.17 ± 0.20 *	4.34 ± 0.12
Q1:0.623 (g/kg/d)	29,923 ± 4344	1.35 ± 0.18 ^ab^	1.17 ± 0.48 ^ab^	4.63 ± 0.29 ^bc^
Q2:0.823	31,620 ± 4113	1.27 ± 0.17 ^ab^	1.43 ± 0.45 ^ab^	4.37 ± 0.26 ^bc^
Q3:0.975	34,565 ± 4186	1.32 ± 0.17 ^ab^	1.16 ± 0.46 ^ab^	4.46 ± 0.27 ^bc^
Q4:1.182	19,404 ± 3978	1.05 ± 0.16 ^a^	1.11 ± 0.44 ^ab^	4.05 ± 0.27 ^bc^
Q5:1.518	17,979 ± 3593	1.22 ± 0.15 ^ab^	0.99 ± 0.40 ^a^	4.26 ± 0.24 ^bc^
Women ≥ 60 years (overall)	33,343 ± 2652	1.68 ± 0.11 **	3.46 ± 0.29 **	4.10 ± 0.18
Q1:0.623 (g/kg/d)	43,978 ± 6143	1.40 ± 0.25 ^b^	3.24 ± 0.66 ^c^	4.14 ± 0.41 ^bc^
Q2:0.823	43,290 ± 7004	2.13 ± 0.28 ^c^	2.16 ± 0.77 ^bc^	4.48 ± 0.46 ^bc^
Q3:0.975	30,259 ± 5081	1.54 ± 0.21 ^b^	1.6 ± 0.56 ^bc^	4.53 ± 0.35 ^bc^
Q4:1.182	25,625 ± 5221	1.61 ± 0.21 ^bc^	2.97 ± 0.57 ^c^	3.58 ± 0.21 ^a^
Q5:1.518	23,560 ± 5919	1.72 ± 0.24 ^bc^	7.35 ± 0.65 ^d^	3.77 ± 0.39 ^ab^

Three Way ANOVA ^abcd^: significant differences (*p* < 0.05) between different letters by quintile; (* vs. **) by age.

**Table 3:** Pearson correlation matrix.

	BMI (kg/m^2^)	Age (Years)	Leptin (pg/mL)	TNF-α (pg/mL)	IL-6 (pg/mL)	IL-15 (pg/mL)
AP (g/d/kg)	−0.228					
	*p* < 0.001	n.s.	n.s.	n.s.	n.s.	n.s.
PP (g/d/kg)	−0.489	−0.114	−0.235	−0.119		
	*p* < 0.001	*p* = 0.034	*p* < 0.001	*p* = 0.027	n.s.	n.s.
Fibre (g/d)	−0.160		−0.162			
	*p* = 0.003	n.s.	*p* = 0.003	n.s.	n.s.	n.s.
Leptin (pg/mL)	0.535			0.123	0.137	
	*p* < 0.001	n.s.		*p* = 0.022	*p* = 0.011	n.s.
TNF-α (pg/mL)	0.161	0.301	0.123		0.224	
	*p* = 0.003	<0.001	*p* = 0.022		*p* < 0.001	n.s.
IL-6 (pg/mL)		0.341	0.137	0.224		
	n.s.	<0.001	*p* = 0.011	*p* < 0.001		n.s.
IL-15 (pg/mL)		0.135				
	n.s.	*p* = 0.012	n.s.	n.s.	n.s.	

n.s. = not significant.

## Data Availability

The data presented in this study are available on request from the first author due to privacy and/or ethical restrictions.

## Supplementary Materials

The following supporting information can be downloaded at: https://www.mdpi.com/article/10.3390/ijms23148008/s1.

## References

[1] GBD (2020). 2019 Disease and Injuries Collaborators Global burden of 369 diseases and injuries in 204 countries and territories, 1990–2019: A systematic analysis for the Global Burden of Disease Study 2019. Lancet.

[2] Silveira B.K.S., Oliveira T.M.S., Andrade P.A., Hermsdorff H.H.M., Rosa C.O.B., Franceschini S.D.C.C. (2018). Dietary Pattern and Macronutrients Profile on the Variation of Inflammatory Biomarkers: Scientific Update. Cardiol. Res. Pract..

[3] Bajaj V., Gadi N., Spihlman A.P., Wu S.C., Choi C.H., Moulton V.R. (2021). Aging, Immunity, and COVID-19: How Age Influences the Host Immune Response to Coronavirus Infections?. Front. Physiol..

[4] Li P., Yin Y.L., Li D., Kim S.W., Wu G. (2007). Amino acids and immune function. Br. J. Nutr..

[5] Lonnie M., Hooker E., Brunstrom J.M., Corfe B.M., Green M.A., Watson A.W., Williams E.A., Stevenson E.J., Penson S., Johnstone A.M. (2018). Protein for Life: Review of Optimal Protein Intake. Sustainable Dietary Sources and the Effect on Appetite in Ageing Adults. Nutrients.

[6] Song M., Fung T.T., Hu F.B., Willett W.C., Longo V.D., Chan A.T., Giovannucci E.L. (2016). Association of Animal and Plant Protein Intake with All-Cause and Cause-Specific Mortality. JAMA.

[7] Azzini E., Barnaba L., Intorre F., Ciarapica D., Verrascina M., Zanetti B., Venneria E., Foddai M.S., Maiani F., Monteleone M. (2020). Food System Dynamics in Rural Environments and Health Benefits of the Mediterranean Diet. Int. J. Clin. Nutr. Diet..

[8] Polito A., Azzini E., Barnaba L., Verrascina M., Zanetti B., Monteleone A., Intorre F., Ciarapica D., Tomassini S., Guidarelli L. (2020). Socio-economic drivers in productive rural activities and their impact on the eating habits. lifestyle and nutritional status of people living in a rural area: The Majella National Park as a case study. Econ. Agr.-Aliment..

[9] EFSA NDA Panel (EFSA Panel on Dietetic Products (2012). Nutrition and Allergies). Scientific Opinion on Dietary Reference Values for protein. EFSA J..

[10] Willett W., Rockström J., Loken B., Springmann M., Lang T., Vermeulen S., Garnett T., Tilman D., DeClerck F., Wood A. (2019). Food in the Anthropocene: The EAT-Lancet Commission on healthy diets from sustainable food systems. Lancet.

[11] Montalcini T., Pujia A., Donini L.M., Frittitta L., Galvano F., Natali A., Pironi L., Porrini M., Riso P., Rivellese A.A. (2020). A Call to Action: Now Is the Time to Screen Elderly and Treat Osteo-sarcopenia. A Position Paper of the Italian College of Academic Nutritionists MED/49 (ICAN-49). Nutrients.

[12] Hertzler S.R., Lieblein-Boff J.C., Weiler M., Allgeier C. (2020). Plant Proteins: Assessing Their Nutritional Quality and Effects on Health and Physical Function. Nutrients.

[13] Pellinen T., Päivärinta E., Isotalo J., Lehtovirta M., Itkonen S.T., Korkalo L., Erkkola M., Pajari A.M. (2022). Replacing dietary animal-source proteins with plant-source proteins changes dietary intake and status of vitamins and minerals in healthy adults: A 12-week randomized controlled trial. Eur. J. Nutr..

[14] Li P., Wu G. (2022). Important roles of amino acids in immune responses. Br. J. Nutr..

[15] Hart M.J., Torres S.J., McNaughton S.A., Milte C.M. (2021). Dietary patterns and associations with biomarkers of inflammation in adults: A systematic review of observational studies. Nutr. J..

[16] Coelho-Júnior H.J., Trichopoulou A., Panza F. (2021). Cross-sectional and longitudinal as-sociations between adherence to Mediterranean diet with physical performance and cognitive function in older adults: A systematic review and meta-analysis. Ageing Res. Rev..

[17] Aleksandrova K., Koelman L., Rodrigues C.E. (2021). Dietary patterns and biomarkers of oxidative stress and inflammation: A systematic review of observational and intervention studies. Redox Biol..

[18] Hassanzadeh-Rostami Z., Faghih S. (2021). Effect of Dietary Fiber on Serum Leptin Level: A Systematic Review and Meta-Analysis of Randomized Controlled Trials. Exp. Clin. Endocrinol. Diabetes.

[19] Zhubi-Bakija F., Bajraktari G., Bytyçi I., Mikhailidis D.P., Henein M.Y., Latkovskis G., Rexhaj Z., Zhubi E., Banach M. (2021). International Lipid Expert Panel (ILEP). The impact of type of dietary protein. animal versus vegetable. in modifying cardiometabolic risk factors: A position paper from the International Lipid Expert Panel (ILEP). Clin. Nutr..

[20] Chung H.Y., Kim D.H., Lee E.K., Chung K.W., Chung S., Lee B., Seo A.Y., Chung J.H., Jung Y.S., Im E. (2019). Redefining Chronic Inflammation in Aging and Age-Related Diseases: Proposal of the Senoinflammation Concept. Aging Dis..

[21] Franceschi C., Capri M., Garagnani P., Ostan R., Santoro A., Monti D., Salvioli S., Fulop T., Franceschi C., Hirokawa K., Pawelec G. (2019). Inflammaging. Handbook of Immunosenescence.

[22] Ferrucci L., Fabbri E. (2018). Inflammageing: Chronic inflammation in aging. cardiovascular disease, and frailty. Nat. Rev. Cardiol..

[23] Leitão C., Mignano A., Estrela M., Fardilha M., Figueiras A., Roqu F., Her-deiro M.T. (2022). The Effect of Nutrition on Aging—A Systematic Review Focusing on Aging-Related Biomarkers. Nutrients.

[24] Simonson M., Boirie Y., Guillet C. (2020). Protein. amino acids and obesity treatment. Rev. Endocr. Metab. Disord..

[25] Day L., Cakebread J.A., Loveday S.M. (2022). Food proteins from animals and plants: Differences in the nutritional and functional properties. Trends Food Sci. Technol..

[26] Branca F., Lartey A., Oenema S., Aguayo V., Stordalen G.A., Richardson R., Mario A., Ashkan A. (2019). Transforming the food system to fight non-communicable diseases. BMJ.

[27] Gayathri R., Ruchi V., Mohan V. (1999). DietoMetro—Indagine Alimentare Con Fotografie.

[28] Italian Food Composition Tables. https://www.crea.gov.it/-/tabella-di-composizione-degli-alimenti.

[29] LARN SINU IV Revisione dei Livelli di Assunzione di Riferimento di Nutrienti ed Energia per la Popolazione Italiana 2014. https://sinu.it/larn/.

[30] Lohman T.G., Roche A.F., Martorell R. (1988). Anthropometric Standardization Reference Manual.

[31] WHO (1995). Physical Status: The Use and Interpretation of Anthropometry.

[32] Durnin J.V.G.A. (1974). Womersley, Body fat assessed from total body density and its estimation from skinfold thickness: Measurements on 481 men and women aged from 16 to 72 years. Br. J. Nutr..

[33] Brozek J., Henschel A. (1961). Siri WE Body composition from fluid spaces and density: Analysis of methods. Techniques for Measuring Body Composition.

[34] Wallace T.M., Levy J.C., Matthews D.R. (2004). Use and abuse of HOMA modeling. Diabetes Care..

[35] Muniyappa R., Lee S., Chen H., Quon M.J. (2008). Current approaches for assessing insulin sensitivity and resistance in vivo: Advantages. limitations. and appropriate usage. Am. J. Physiol. Endocrinol. Metab..

[36] NHLBI. Obesity Education Initiative Expert Panel on the Identification, Evaluation, and Treatment of Obesity in Adults (US) Clinical Guidelines on the Identification, Evaluation, and Treatment of Overweight and Obesity in Adults: The Evidence Report. Bethesda (MD): National Heart, Lung, and Blood Institute; September 1998 Table IV-2. Classification of Overweight and Obesity by BMI. Waist Circumference and Associated Disease Risk*. https://www.ncbi.nlm.nih.gov/books/NBK2004/table/A242/.

[37] Lam D.W., LeRoith D., Feingold K.R., Anawalt B., Boyce A., Chrousos G., de Herder W.W., Dhatariya K., Dungan K., Hershman J.M., Hofland J., Kalra S. (2019). Metabolic Syndrome. Endotext.

[38] Elkind M., Albert M., Lloyd-Jones D.M. (2022). Road to Equity in Brain Health. Circulation.

[39] Flack J.M., Adekola B. (2020). Blood pressure and the new ACC/AHA hypertension guidelines. Trends Cardiovasc. Med..

