# Supplementary material for: Total and Plant Protein Consumption: The Role of Inflammation and Risk of Non-Communicable Disease
Source: Int J Mol Sci. 2022 Jul 20;23(14):8008. doi: 10.3390/ijms23148008 (PMC9318066; doi:10.3390/ijms23148008)
Supplement: Supplementary file 1 [file ijms-23-08008-s001.zip › ijms-1821105-supplementary.pdf]

Total and Plant Protein Consumption: the role on inflammation and Non-Communicable Disease Risk Elena Azzini, Ilaria Peluso, Federica Intorre, Lorenzo Barnaba, Eugenia Venneria, Maria Stella Foddai, Donatella Ciarapica, Francesca Maiani, Anna Raguzzini and Angela Polito

CREA-Research Centre for Food and Nutrition, Via Ardeatina 546, 00178 Rome, Italy

Table S1. Physical characteristics and HOMA-IR by sex, class age and quintile category of average protein intake (g/kg/d)

Quintiles of protein intake		AGE years		BMI kg/m^2		FAT kg		FM %		FFM kg		HOMA-IR	
by sex and age	n	Mean	Sd	mean	sd	mean	sd	mean	sd	mean	sd	mean	sem
MEN	119	57,7	15,9	27,34	0,42	56,4	56,4	28,36	0,50^{a}	56,4	6,7	2,81	0,24
Men <60 years (overall)	68	45,1	11,4	26,7	3,9	22,1	6,7	27,1	5,9	57,9	7,4	2,51	0,29*
Q1:0.623 (g/kg/d)	15	47,2	10,9	29,5	4,7 ^{a,b}	25,4	6,4 ^{a}	29,1	4,6	61,7	10,7	4,18	0,60
Q2:0.823	16	48,8	7,1	27,7	3,2	24,8	6,1 ^{b}	29,9	4,6^{a}	57,3	5,7	2,35	0,58
Q3:0.975	11	45,5	13,3	26,2	3,2	22,7	6,4	28,1	6,2	57,1	6,5	1,89	0,74
Q4:1.182	15	39,9	12,6	25,5	3,2 ^{a}	19,5	6.9	24,4	6,4	59,0	5,1	2,34	0,62
Q5:1.518	11	43,7	12,5	23,9	2,4 ^{b}	16,4	5,6 ^{a,b}	23,2	5,9^{a}	53,2	5,5	1,80	0,70
Men ≥60 years (overall)	51	70,4	6,9	28,2	3,3	23,7	6,0	30,0	4,8	54,3	4,9	3,12	0,37**
Q1:0.623 (g/kg/d)	14	72,6	7,0	28,8	3,9	24,1	6,7	29,7	4,8	55,6	5,3	2,66	0,64
Q2:0.823	14	68,4	7,5	27,9	2,7	23,9	4,	30,4	4,0	54,1	5,1	3,11	0,62
Q3:0.975	11	69,8	6,3	28,8	3,2	25,8	6,7	31,6	6,0	54,9	3,4	5,04	0,70
Q4:1.182	5	70,6	5,7	27,9	3,2	21,4	5,6	27,8	4,9	54,7	3,7	1,23	1,16
Q5:1.518	7	70,6	7,3	27,4	4,1	21,1	5,9	29,0	4,5	50,8	6,4	3,56	0,88
WOMEN	228	57,41	15,2	27,90	0,31	41,5	41,5	38,17	0,37^{b}	41,5	6,4	2.45	0,17
Women <60 years (overall)	153	45,23	11,6	26,9	0,35*	41,6	41,6	37,45	0,42**	41,6	6,8	2,11	0,19
Q1:0.623 (g/kg/d)	26	46,5	11,2	29,1	6,6^{c}	29,7	9,3 ^{a,b}	39,3	5,1^{a}	44,6	7,8	2,32	0,64
Q2:0.823	30	48,7	9,6	29,9	6,2 ^{d,e}	29,7	8,9 ^{c,d}	39,2	6,2 ^{b}	44,9	8,5	2,37	0,43
Q3:0.975	28	45,2	12,2	26,8	3,7	26,0	6,8 ^{e}	38,2	5,0	41,3	4,7	2,26	0,44
Q4:1.182	31	42,5	12,3	25,5	4,4 ^{d}	23,2	6,3 ^{a,c}	35,4	4,8	41,4	5,8	1,67	0,42
Q5:1.518	38	43,9	12,2	23,7	3,5 ^{c,e}	20,6	5,3^{b,d,e}	35,0	5,6^{a,b}	37,4	3,7	1,90	0,38
Women ≥60 years (overall)	75	69,5	5,7	28,8	4,7	26,7	6,5	38,9	4,5	41,3	5,7	2,79	0,28
Q1:0.623 (g/kg/d)	13	69,8	5,6	30,4	4,4 ^{f}	29,1	7,4	40,2	5,7	42,6	4,3 ^{a,b}	3,77	0,64
Q2:0.823	10	68,0	5,7	31,5	4,3 ^{g}	29,5	6,3	39,1	5,0	45,3	5,0 ^{c,d}	3,93	0,73
Q3:0.975	19	69,1	4,7	30,2	4,7 ^{h}	28,4	6,8	39,7	4,0	42,5	5,9 ^{e}	1,89	0,55
Q4:1.182	19	69,7	5,7	27,0	4,1	24,9	5,1	38,5	3,5	39,5	5,3 ^{a,c}	2,48	0,53
Q5:1.518	14	70,9	7,3	24,9	2,5 ^{f,g,h}	22,6	5,0	37,0	4,9	38,1	5,3 ^{b,d,e}	1,88	0,62
**P-Value§**													
**Sex**		n.s.		n.s		0,000		0,000		0,000		n.s.	
**Age class**		0,000		0,001		n.s.		0,001		0,007		0,026	
**Quintile**		n.s		0.000		0,000		0,000		0,000		0,046	
**Interaction**													
**Sex * Age class**		n.s		n.s		n.s		n.s		n.s		n.s.	
**Sex *Quintile**		n.s		n.s		n.s		n.s		n.s		n.s.	
**Age class *Quintile**		n.s		n.s		n.s		n.s		n.s		n.s.	
**Sex*Age class*Quintile**		n.s		n.s		n.s		n.s		n.s		0,001	

§Three Way Analysis of Variance Factor: The values with different superscript letters in the same column are significantly different ($p<0.05$). Three Way Analysis of Variance Factor by sex (a *vs* b) by class age (* *vs***), by quintiles (^{abcdefgh}) BMI = body mass index, FFM= Fat free mass; FAT= fat mass, HOMA-IR= Homeostatic Model Assessment for Insulin Resistance

Total and Plant Protein Consumption: the role on inflammation and Non-Communicable Disease Risk Elena Azzini, Ilaria Peluso, Federica Intorre, Lorenzo Barnaba, Eugenia Venneria, Maria Stella Foddai, Donatella Ciarapica, Francesca Maiani, Anna Raguzzini and Angela Polito
CREA-Research Centre for Food and Nutrition, Via Ardeatina 546, 00178 Rome, Italy

Table S2. Prevalence (%) of disease risk factors by sex, class age and quintile category of average protein intake (g/kgxd)

	**Healthy †**		**Medium NCDs risk †**		**High NCDs risk †**		**Hypertension †**		**Dyslipidemia †**		**MetSyn †**	
	n	%	n	%	n	%	n	%	N	%	n	%
Men <60 years (overall)	12	18	29	43	16	24	24	35[a*]	14	21	32	47
Q1:0.623 (g/kg/d)	1	8	5	17	7	44	7	29	1	7	12	38
Q2:0.823			10	34	3	19	5	21	6	43	10	31
Q3:0.975	2	17	5	17	2	13	3	13	3	21	3	9
Q4:1.182	4	33	5	17	3	19	6	25	2	14	4	13
Q5:1.518	5	42	4	14	1	6	3	13	2	14	3	9
Men ≥60 years (overall)	1	2	23	45	20	39	43	84[a**]	24	47	39	76
Q1:0.623 (g/kg/d)			8	35	5	25	13	30	5	21	11	28
Q2:0.823			8	35	4	20	12	28	7	29	13	33
Q3:0.975			4	17	6	30	7	16	4	17	7	18
Q4:1.182	1	100	2	9	2	10	4	9	4	17	3	8
Q5:1.518			1	4	3	15	7	16	4	17	5	13
Women <60 years (overall)	36	24	40	26	48	31	36	24[b*]	26	17	79	52
Q1:0.623 (g/kg/d)	2	6	5	13	13	27	7	19	9	35	18	23
Q2:0.823	6	17	3	8	20	42	8	22	6	23	17	22
Q3:0.975	4	11	14	35	7	15	7	19	3	12	16	20
Q4:1.182	9	25	11	28	4	8	8	22	2	8	13	16
Q5:1.518	15	42	7	18	4	8	6	17	6	23	15	19
Women ≥60 years (overall)	2	3	16	21	41	55	55	73[b **]	38	51	57	76
Q1:0.623 (g/kg/d)	1	50	3	19	9	22	8	15	7	18	10	18
Q2:0.823			1	6	8	20	8	15	4	11	6	11
Q3:0.975			5	31	11	27	13	24	10	26	13	23
Q4:1.182			5	31	9	22	14	25	11	29	18	32
Q5:1.518	1	50	2	13	4	10	12	22	6	16	10	18

Prevalence of hypertension ($p<0.05$) was significantly higher among men than women (a vs b). Prevalence of CV risk, metabolic syndrome, hypertension, and dyslipidaemia was significantly ($P<0.05$) higher in older subjects (* vs **). † Differences in health outcomes and quintiles explored by $\chi 2$ test: CV risk (P = <0,001); Hypertension (P = 0,002); MetSyn (P = <0,001); Dyslipidemia (P = 0,003)
